# Dual synergistic role of phosphorylated microcrystalline cellulose as organoinitiator and filler for PCL biocomposites

**DOI:** 10.1039/d6ra07997k

**Published:** 2026-09-30

**Authors:** Hassan Ait Yachou, El Hassan Boutriouia, Taha El Assimi, Sara Blilid, Abderrahim Maazouz, Hicham Ben Youcef, Khalid Lamnawar, Abdelkrim El Kadib, Mohammed Lahcini

**Affiliations:** a IMED-Lab, Faculty of Sciences and Techniques, Cadi Ayyad University (UCA) Avenue Abdelkrim Elkhattabi, B.P 549 40000 Marrakech Morocco m.lahcini@uca.ac.ma; b Univ Lyon, CNRS, UMR 5223, Ingénierie des Matériaux Polymères, INSA Lyon, Université Claude Bernard Lyon 1, Université Jean-Monnet F-69621 Villeurbanne France; c College of Chemical Sciences and Engineering (CCSE), Mohammed VI Polytechnic University (UM6P) Benguerir 43150 Morocco; d Euro-Med University of Fes (UEMF) Route de Meknès, Rond-point de Bensouda Fez 30070 Morocco; e Hassan II Academy of Science and Technology Rabat Morocco

## Abstract

In the present work, phosphorylated microcrystalline cellulose MCC@P was successfully prepared using phosphorus oxychloride (POCl_3_) and employed both as an eco-friendly metal-free initiator for the ring-opening polymerization (ROP) of ε-caprolactone (ε-CL) and as an organic functional filler for the resulting biocomposites. Poly(ε-caprolactone) (PCL) biocomposites were prepared under solvent-free conditions without the use of any metal catalyst. In this system, phosphorylated MCC serves a dual role. The phosphate groups associated with MCC participate in the initiation of ε-CL polymerization, while the remaining hydroxyl groups may serve as potential initiation and grafting sites, leading to the formation of MCC@P-g-PCL biocomposites. The prepared biocomposites were fully characterized using (^31^P/^13^C) nuclear magnetic resonance (NMR), Fourier transform infrared (FTIR), X-ray diffraction (XRD) and dynamic light scattering (DLS), and a suspension stability test to investigate the formation of the MCC@P-g-PCL biocomposites and provide complementary evidence supporting the proposed grafting of PCL onto MCC@P. The molecular-weight characteristics of the extracted free PCL fraction were determined by SEC, with *M*_*n*_ values ranging from 8312 to 21 880 g mol^−1^ depending on the MCC@P content, while thermal stability of the prepared biocomposites was significantly enhanced compared to the neat PCL as demonstrated by the differential scanning calorimetry (DSC) and thermogravimetric analysis (TGA). For instance, the thermal degradation temperature at 30% (*T*_d30%_) weight loss of MCC@P-g-PCL 1% reached 394 °C instead of 372 °C for PCL used as standard. The DSC results showed higher apparent crystallinity values for the MCC@P-g-PCL biocomposites than for neat PCL, with the highest value obtained for MCC@P-g-PCL 1% (64.8% *versus* 52.1% for neat PCL). The heat-release parameters measured by microscale combustion calorimetry were reduced for the MCC@P-g-PCL biocomposites, with the largest reduction in HRR observed for the 3 wt% formulation. Overall, these results highlight the potential of phosphorylated MCC as a metal-free initiating and functional-filler strategy for the development of PCL-based biocomposites with modified thermal and heat-release behavior.

## Abstract 2

Phosphorylated microcrystalline cellulose (MCC@P) was successfully prepared using POCl_3_ and used as an eco-friendly metal-free initiator for the ring-opening polymerization (ROP) and as an organic functional filler for the resulting biocomposites.

## Graphical abstract

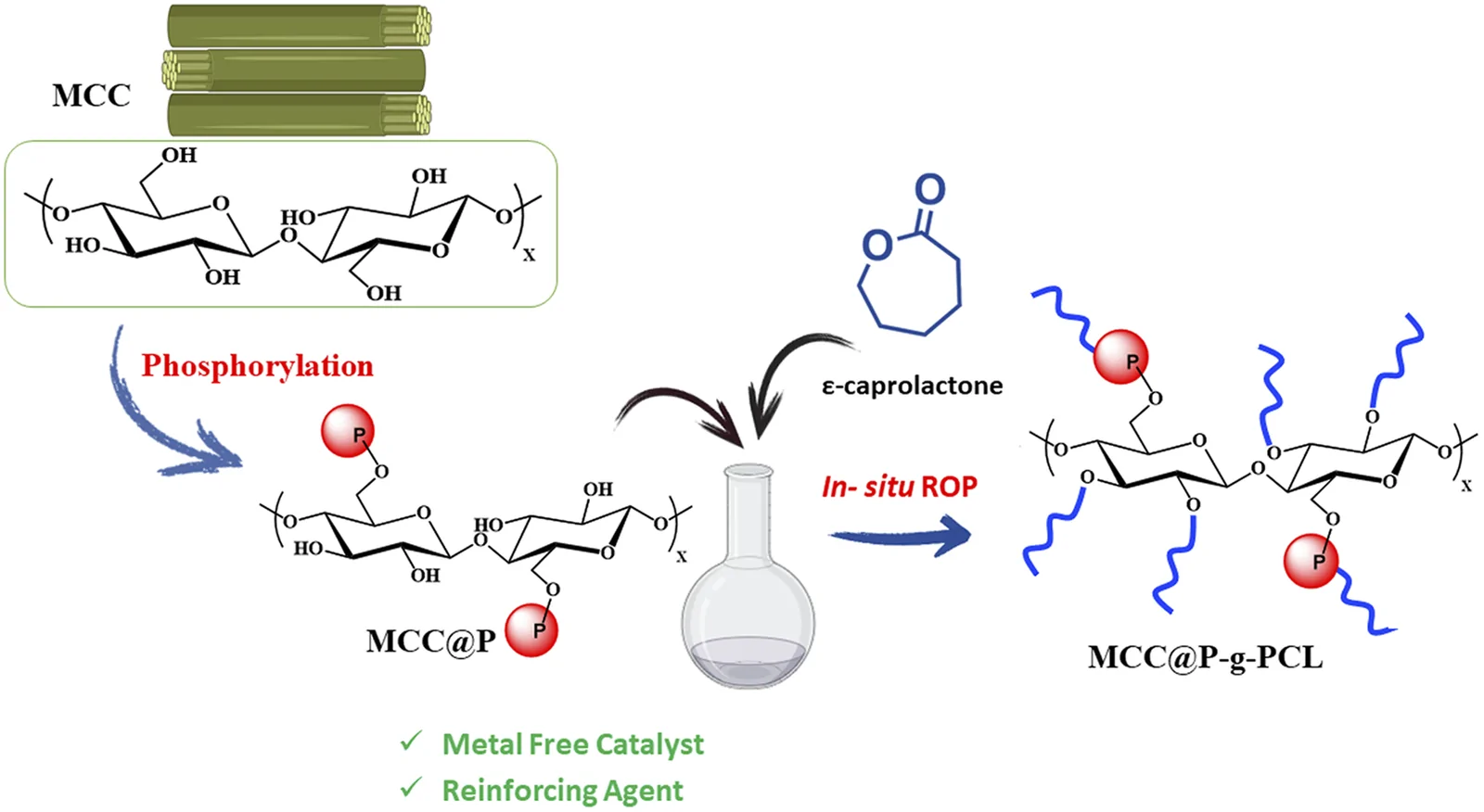

## 1 Introduction

The escalating environmental crisis caused by plastic pollution demands urgent attention. Projections indicate a staggering accumulation of approximately 12 000 million tons of non-biodegradable polymers by 2050 if no decisive actions are taken.^1^ This immense buildup of persistent plastics presents severe challenges, posing significant threats to both the environment and human health, including widespread disruptions to wildlife, pervasive microplastic contamination, and the release of harmful chemical pollutants.^2^

Consequently, a critical global imperative has emerged: to accelerate the development of innovative, biodegradable, and environmentally benign plastic materials.

In this crucial pursuit, aliphatic polyesters have emerged since the late 20th century as a highly promising and viable substitute for conventional, non-biodegradable plastics prevalent in the market.^3^ Their appeal stems from inherent advantages such as biodegradability, sustainability, lower carbon emissions, and low toxicity, making them versatile for diverse applications.^4^

However, despite these merits, native aliphatic polyesters possess intrinsic limitations, notably their restricted thermal and mechanical performance, which significantly curtails their utility in critical sectors such as packaging. Furthermore, their pronounced hydrophobic nature presents a second major hurdle, impeding their broader adoption in applications requiring good surface wettability, such as water treatment, filtration, biomedical devices, tissue engineering, and agriculture.^5^

To overcome these inherent shortcomings, aliphatic polyesters have been strategically formulated into composite materials by incorporating reinforcing agents like fibers or nanofillers within the polymer matrix.^6–8^ Polysaccharides, recognized for their sustainable attributes, have been effectively utilized as fillers within aliphatic polyester matrices, preserving biodegradability while concurrently enhancing physicochemical properties.^9–14^ Within this context, cellulose, as the most abundant polysaccharide, has been extensively integrated into aliphatic polyester matrices using various methodologies. It is important to note that the conventional melt blending method, involving the physical mixing of hydrophobic aliphatic polyester with hydrophilic fillers like cellulose, frequently leads to difficulties in achieving a uniform distribution of the filler throughout the polymer matrix.^15^ Conversely, grafting methods, whether ‘grafting from’ or ‘grafting to’ offer an effective approach by chemically attaching polymer chains (aliphatic polyester) directly onto the cellulose substrate. This chemical linkage ensures a highly uniform dispersion of the filler within the polymer matrix, thereby yielding polymer composites with significantly enhanced properties.^16,17^

The ‘grafting from’ technique, in particular, is highly advantageous as it facilitates higher polymer density and precise control over molecular weight by allowing polymerization to be initiate directly from the surface, effectively mitigating steric hindrance and ensuring the uniform growth of polymeric chains.^18^

Poly(ε-caprolactone) (PCL) is a synthetic aliphatic polyester with a notable history in polymer chemistry and industrial applications, widely used in biomedical fields due to its biodegradability and compatibility with human tissues.^19^ PCL continues to find applications in diverse fields, from packaging to 3D printing.^20,21^ PCL is preferably synthesized through ring-opening polymerization (ROP) of ε-caprolactone (ε-CL), which offers precise control over polymer properties, enables the synthesis of high molecular weight polymers with low dispersity, and allows the grafting of specific functional groups onto polymer chain ends.^22^ The coordination–insertion ROP, commonly used for PCL synthesis, involves catalyst coordination to the monomer, facilitating ring-opening and continuous chain growth.^23^ While tin-alkoxides or alkanoates are common catalysts for *in situ* ROP of ε-CL, their toxicity and moisture sensitivity necessitate inert atmospheres.^23^ The use of organocatalysts as a sustainable alternative to metal catalysts has been explored to overcome these drawbacks. In that regard, several research studies have shown that the ROP of cyclic esters can be efficiently performed using organocatalysts such as N-heterocyclic carbene (NHC),^24–26^ 4-(dimethylamino)pyridine,^27^ phosphine,^28^ phosphazene,^29,30^ and diphenyl phosphate.^31^ Our recent work showed that phosphorus pentoxide (P_2_O_5_) acts as an efficient metal-free catalyst for the homopolymerization of ε-CL,^32^ highlighting the potential of phosphorylated compounds as sustainable options for poly(ε-caprolactone) synthesis. We hypothesize that phosphorylated microcrystalline cellulose (MCC@P) can serve a dual, synergistic role as both an eco-friendly, metal-free organoinitiator for the ring-opening polymerization of ε-caprolactone and an effective functional filler. This dual functionality is expected to lead to the formation of novel poly(ε-caprolactone) (PCL) biocomposites with significantly enhanced physicochemical properties, including improved thermal stability and heat-release behavior, while maintaining their inherent biodegradability. In this context, the present study investigates the innovative use of phosphorylated microcrystalline cellulose (MCC@P), pre-functionalized to fulfill this dual role: (i) initiating the ROP of ε-CL through its acidic phosphoryl groups, and (ii) acting as a functional filler to enhance the physicochemical properties of the resulting PCL biocomposite. To the best of our knowledge, this work represents the first exploration of phosphorylated polysaccharides serving simultaneously as both initiators and functional organic filler for the preparation of PCL polysaccharide biocomposites.

The choice of microcrystalline cellulose (MCC) was motivated by the fact that it stands out. Derived from cellulose, a naturally occurring polysaccharide abundant in plant cell walls, MCC is typically produced through controlled acid hydrolysis of cellulose sources such as wood pulp or cotton fibers, a process that selectively isolates crystalline microfibrils by removing amorphous regions.^33^ While MCC finds extensive applications across catalysis,^34^ food, cosmetic, and medical industries,^35^ its significant role as a reinforcing agent in polymer composites is particularly noteworthy. Indeed, MCC is highly favored over conventional reinforcing agents such as glass fiber, silica, and carbon black due to its advantageous properties, including low density, minimal equipment abrasion, inherent renewability, and complete biodegradability.^36^

The synthesis of PCL biocomposite (MCC@P-g-PCL) underwent comprehensive characterization to investigate the synergistic interactions between the polymeric matrix (PCL) and the phosphorylated reinforcement (MCC@P). This study highlights the potential of phosphorylated cellulosic fillers (MCC) in paving the way for the development of high-performance, sustainable materials with enhanced functionality.

## 2 Experimental

### 2.1 Materials

Microcrystalline Cellulose (MCC) (CAS 9004-34-6, (C_6_H_10_O_5_)_*n*_, *n* ≈ 220, molecular weight ≈36 000 Da) was purchased from Sigma-Aldrich. Phosphorus oxychloride (POCl_3_), tetrahydrofuran (THF), methanol (MeOH), dichloromethane (DCM), acetone, ε-caprolactone (ε-CL), and poly(ε-caprolactone) (PCL) 14 000 g mol^−1^ (CAS N 24980-41-4) and calcium hydride (CaH_2_) were purchased from Sigma-Aldrich.

### 2.2 Synthesis methods

#### 2.2.1 Phosphorylation of microcrystalline cellulose (MCC@P)

The phosphorylated microcrystalline cellulose (MCC@P) was carried out according to our previously published procedure,^37^ where 1 ml of POCl_3_ (1.06 mmol) was slowly added to a suspended MCC (200 mg) in 5 ml THF with continuous stirring for 10 min. The resulting mixture was stirred at room temperature (25 °C) for 24 hours. Next, 1 ml of deionized water was added slowly, filtered, and washed with 1 ml of acetone. The obtained powder was dried in an oven at a temperature at 60 °C for 24 h (step 1, Fig. 1).

**Fig. 1:**
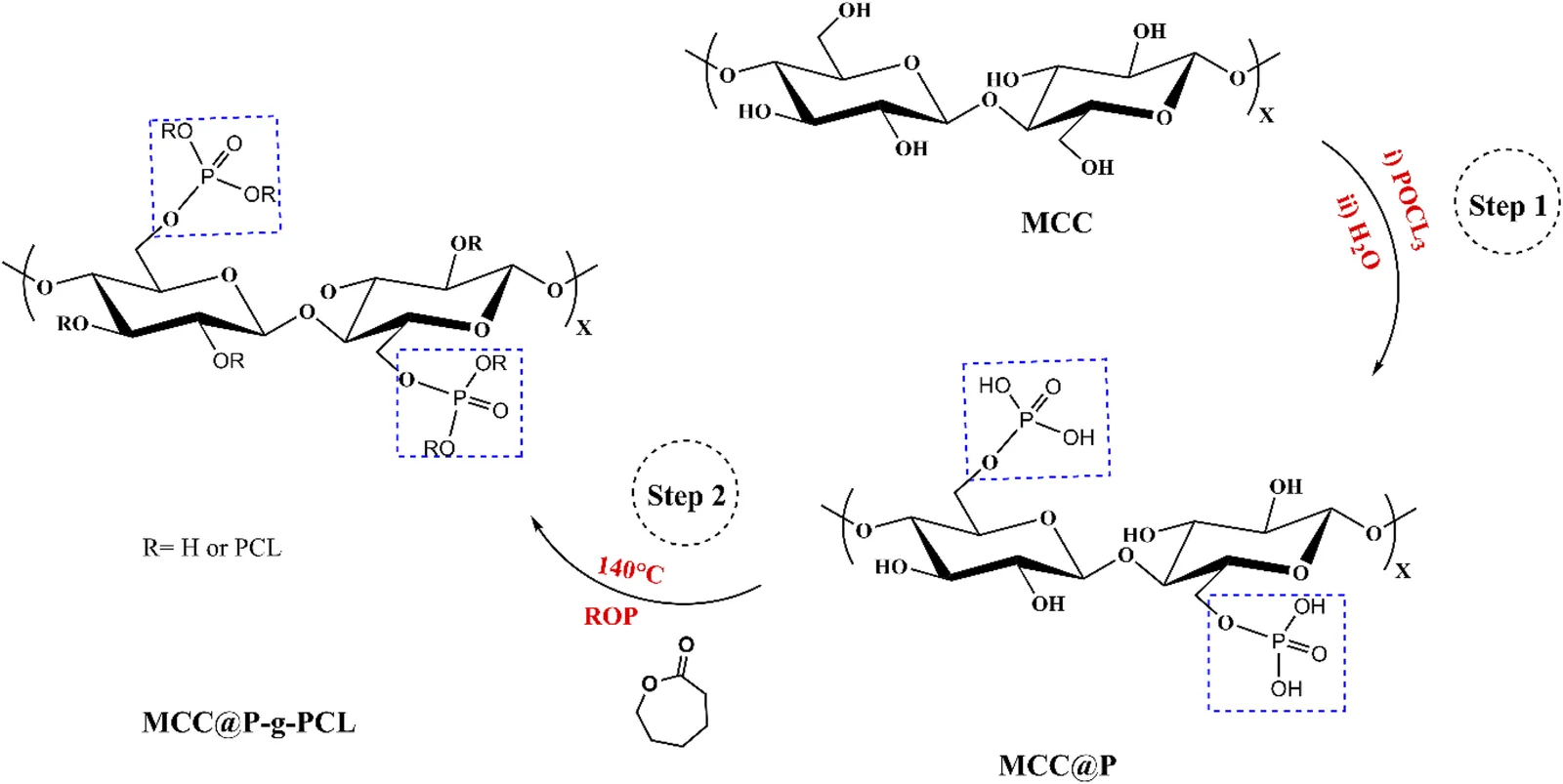
Scheme outlining phosphorylation of microcrystalline cellulose and the synthesis of MCC@P-g-PCL composites.

#### 2.2.2 Synthesis of (MCC@P-g-PCL)

Poly(ε-caprolactone) was grafted on phosphorylated microcrystalline cellulose (MCC@P) *via in situ* ring-opening polymerization of ε-caprolactone (ε-CL). The phosphate groups introduced onto MCC during phosphorylation are considered to participate in the initiation of ε-CL polymerization, while the remaining non-phosphorylated hydroxyl groups are regarded as potential initiation and grafting sites. Before polymerization, the ε-CL monomer was distilled over calcium hydride (CaH_2_) under reduced pressure and stored over molecular sieves (3 Å). The synthetic procedure begins with the dispersion of (20 mg, 60 mg, and 100 mg) phosphorylated MCC in 2 g (17.5 mmol) of monomer liquid (ε-CL) using sonication for 5 minutes at low temperature (0 °C), and then is stirred at room temperature (25 °C) for 1 hour. Afterwards, the resulting mixture was placed into a preheated oil bath (140 °C) for 24 hours in open air to graft PCL chains from MCC@P. It is worth mentioning that several MCC@P/ε-CL weight ratios were investigated, corresponding to MCC@P contents of 1, 3 and 5 wt% to evaluate the effect of MCC@P content on the PCL chains grafted in the resulting (MCC@P-g-PCL) biocomposites.

### 2.3 Free polymer extraction (PCL)

To estimate the approximate surface density of PCL chains on MCC@P, the product was ultrasonicated (ultrasonic bath) in 20 ml THF, then centrifuged at 9000 rpm (×5) for 10 min. The unattached PCL was trapped in the supernatant. The free polymer was precipitated in cold methanol (50 ml) (2 °C), filtered, and dried at 40 °C.^10,38,39^ The grafting yield (*G*_Y_) was calculated gravimetrically following eqn (1) and (2):

1 *m*_(MCC@P-g-PCL)_ = *m*_(MCC@P-g-PCL)+free PCL_ − *m*_Extracted PCL_

2
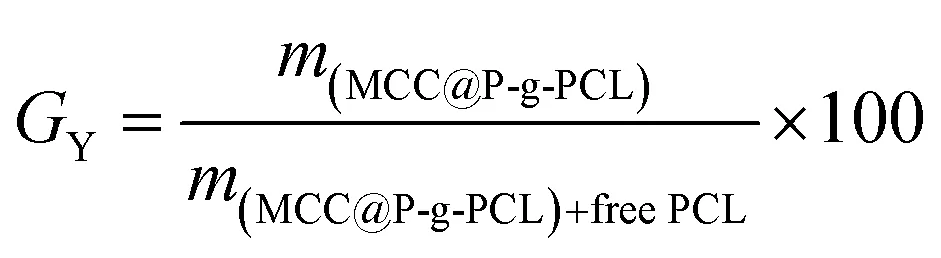
where *m*_MCC@P-g-PCL_ and *m*_Extracted PCL_ are the weights of grafted biocomposite and extracted PCL, respectively. While *m*_(MCC@P-g-PCL)+free PCL_ is the weight of the powder before free PCL extraction.

### 2.4 Characterization methods

#### 2.4.1 Size exclusion chromatography (SEC)

Size exclusion chromatography was investigated to estimate the distribution of the PCL chains' molecular weight using the Agilent Infinity 1260 (II) GPC/SEC equipment. Here, the SEC instrument was connected with a UV (254 nm) sensor and PLgel 5 µm MIXED-C column. The tetrahydrofuran (THF) was used as eluent (1 ml min^−1^) at room temperature. The PLgel 5 µm MIXED-C column was filled with polystyrene-*co*-divinylbenzene (PS-*co*-DVB) copolymer as gel permeation with standardized polystyrene for calibration (PS Kit S-M2-10).

#### 2.4.2 Inductively coupled plasma spectrometer (ICP-OES)

ICP-OES method analyses were performed to quantify the percentages of the phosphorus elements likely to be present on phosphorylated MCC. This measurement, performed after the phosphorylation reaction, enables to determine the degree of substitution (DS) of the phosphate groups from the eqn (3) and the obtained result is illustrated in Table S1 SI. The method was inspired by previous research work.^40^3
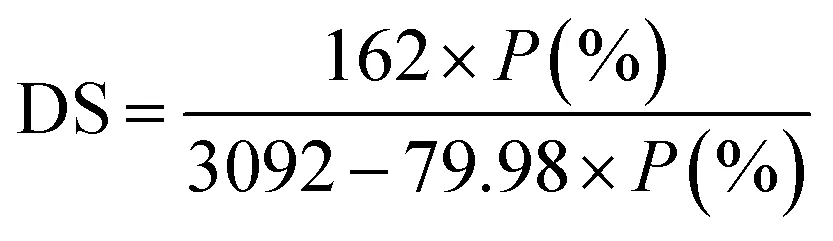
where *P* is the content of % obtained from ICP.

#### 2.4.3 Fourier transform infrared spectroscopy (FTIR)

The FTIR spectra of neat PCL, neat MCC, MCC@P, and MCC@P-g-PCL biocomposites were recorded on a PerkinElmer Spectrum 1000 Fourier transform spectrometer instrument from 500–4000 cm^−1^ at room temperature with a resolution of 4 cm^−1^.

#### 2.4.4 Nuclear magnetic resonance spectroscopy (NMR)

^13^C and ^31^P CP MAS NMR spectra were acquired on a bruker avance 400 WB spectrometer operating at 100 MHz and 162 MHz respectively under cross-polarization conditions.

The phosphorus (^31^P) nuclear magnetic resonance (NMR) spectra have been recorded on Bruker AC 300 spectrometer. CDCl_3_ was used as a deuterated solvent, and chemical shifts were referenced to 85% phosphoric acid (H_3_PO_4_) and tetramethylsilane (TMS) for ^31^P.

#### 2.4.5 Differential scanning calorimetry (DSC)

DSC analysis of MCC@P-g-PCL and neat PCL was performed on a PerkinElmer Jade differential scanning calorimeter under an inert atmosphere (flow of N_2_ and Ar) with a flow rate of 60 ml min^−1^. The samples were heated from 10 °C to 120 °C (10 °C min^−1^) to remove the residues and water traces in the samples and cooled to −60 °C, then heated to 150 °C. Thermal properties were taken from the second heating scan. The percentage of crystallinity (*χ*%) was determined from the following eqn (4):4
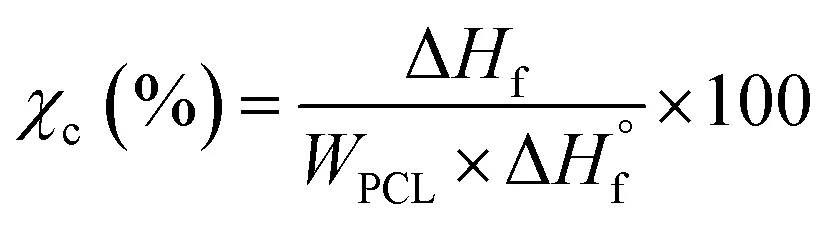


Δ*H*_f_ and 
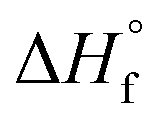
 indicate the melting enthalpy (J g^−1^) calculated from DSC and the theoretical enthalpy value of 100% crystalline polymer (136.4 J g^−1^ for neat PCL),^41^ respectively. *W*_PCL_ presents the weight fraction of PCL in the MCC@P-g-PCL biocomposites.

#### 2.4.6 Thermogravimetric analysis (TGA)

TGA analysis of the neat PCL and MCC@P-g-PCL biocomposites was performed on 55 discovery TA instruments. The samples prepared were heated from room temperature (30 °C) to 600 °C under an argon atmosphere (60 ml min^−1^) with a heating rate of 10 °C min^−1^.

#### 2.4.7 Suspension stability

Suspension stability was carried out to investigate the grafting success of the PCL chains from the phosphorylated microcrystalline cellulose (MCC@P) substrate. MCC@P, the physical mixture of PCL and MCC@P (PCL/MCC@P), and the prepared biocomposite MCC@P-g-PCL were suspended in separate tubes of dichloromethane (DCM) and rigorously stirred at room temperature (25 °C), then the resulting suspensions were compared after 2 h.

#### 2.4.8 X-ray diffraction (XRD)

X-ray diffraction patterns have been carried out on the neat MCC, MCC@P, neat PCL and MCC@P-g-PCL biocomposites using a Bruker D8 Advance diffractometer with monochromatic Cu Kα source operated at 40 kV and 40 mA. The XRD patterns were recorded over a wider angular range, 10–80, (2*θ*) to determine the presence of crystalline phases.

#### 2.4.9 Scanning electron microscopy (SEM)

The morphologies of the MCC, MCC@P, neat PCL, and PCL biocomposites (MCC@P-g-PCL) were observed by scanning electron microscopy using a Vega 3 Tescan scanning electron microscope with conventional tungsten, heated cathode intended both for vacuum and for low vacuum operations. All the samples were previously sputter-coated with Au–Pd in argon plasma to enhance their conductivity.

#### 2.4.10 Dynamical light scattering (DLS)

Particle size distribution was determined using a Malvern Zetasizer Pro with a size range of 0.3 nm to 10 µm. Samples of MCC@P and prepared biocomposites MCC@P-g-PCL were dispersed in THF using an ultrasound bath before analysis. Three measurements were carried out and averaged for each sample.

#### 2.4.11 Microscale combustion calorimeter

Microscale combustion calorimeter measurements were performed using an FTT Micro Calorimeter (Fire Testing Technology Limited, East Grinstead, UK) according to (ASTM) D7309 standard test method. The studied samples, weighing 10 mg, were placed in an alumina ceramic crucible and heated to 700 °C at a heating rate of 1 °C s^−1^ in the pyrolysis chamber under a nitrogen stream. The combustor furnace was set at 900 °C for complete oxidation of the volatilized decomposition products while exposed to a stable nitrogen and oxygen flow rate of 80 and 20 cm^3^ min^−1^, respectively.

## 3 Results and discussion

### 3.1 Synthesis of MCC@P-g-PCL biocomposite prepared

In this study, microcrystalline cellulose MCC was phosphorylated to serve dually as a functional filler and as a metal-free initiator for the *in situ* ring-opening polymerization (ROP) of ε-CL. The functionalization of MCC was carried out using phosphorus oxychloride (POCl_3_) in THF as a suitable solvent at room temperature, ensuring the effective incorporation of phosphate groups onto the cellulose backbone. The phosphorylated microcrystalline cellulose (MCC@P) was evaluated as an initiator in the bulk ring-opening polymerization (ROP) of ε-caprolactone (ε-CL) under solvent-free conditions. To confirm the catalytic role of the grafted phosphorus, a control experiment was first conducted using non-phosphorylated MCC under identical conditions. No polymer formation was observed, highlighting the essential role of phosphorylation in enabling catalytic activity. Following this validation, the effect of initiator loading was investigated. Three different amounts of phosphorylated MCC (MCC@P) (1, 3, and 5 wt%) were tested to evaluate the grafting yield (*G*_Y_) and their influence on the number-average molecular weight (*M*_*n*_) of poly(ε-caprolactone) (PCL) grafted from the MCC@P surface. The synthesis pathway starts by dispersing MCC@P into ε-CL liquid monomer at room temperature, followed by bulk graft polymerization carried out at 140 °C under solvent-free conditions. Both the phosphoryl and hydroxyl functional groups present on MCC@P were actively involved in the ROP of ε-CL, acting respectively as initiator and co-initiator. In that regard, poly(ε-caprolactone) chains were successfully grafted from the surface of phosphorylated microcrystalline cellulose, resulting in the formation of PCL-based biocomposites (MCC@P-g-PCL). Ring-opening polymerization (ROP) of cyclic esters, catalyzed by various Brønsted acids as non-metallic catalysts, has been extensively investigated and well documented in the literature.^31,42–49^ Based on these established mechanistic principles, a mechanistic pathway is proposed for the ROP of ε-CL catalyzed by phosphorylated microcrystalline cellulose (MCC@P), as illustrated in Fig. 2. The acidic phosphate groups introduced onto the MCC backbone activate the ε-CL monomer through interaction of the acidic P–OH groups with the carbonyl group, thereby facilitating ring opening. The activated monomer can then undergo nucleophilic attack by either a phosphate hydroxyl group (P–OH) or a residual hydroxyl group of the cellulose backbone, resulting in ring opening and formation of an ester linkage. Subsequent incorporation of ε-CL units leads to the growth of PCL chains from the MCC@P surface. Accordingly, the proposed pathway involves the formation of PCL chains through ester linkages at the phosphorylated sites and/or at residual hydroxyl groups of the cellulose backbone, ultimately resulting in the formation of MCC@P-g-PCL biocomposites. A similar catalytic mechanism has been previously reported in the ROP using phosphorus-based catalyst.^44–46^

**Fig. 2:**
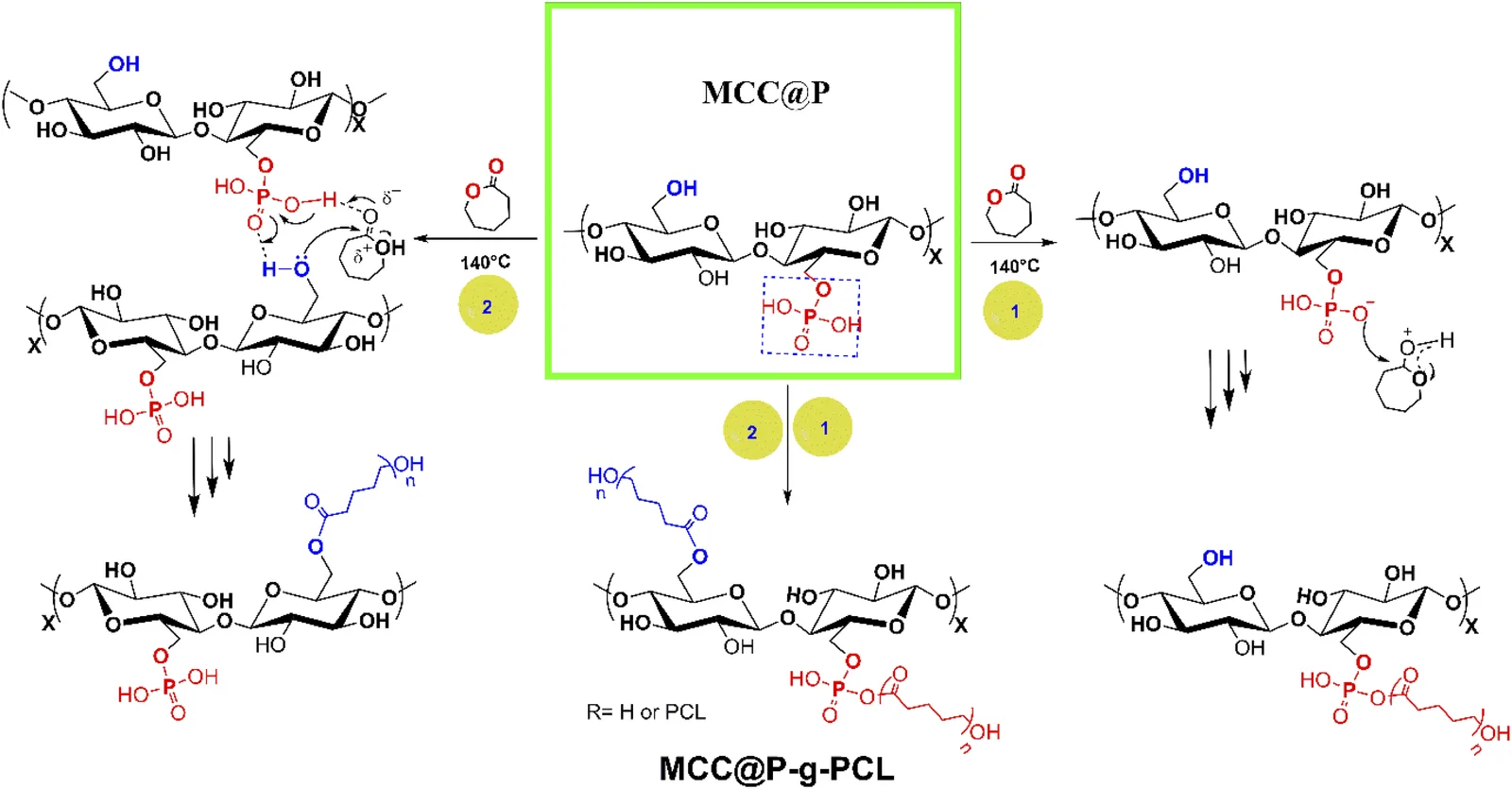
Proposed mechanism for ROP of ε-CL from MCC@P.

### 3.2 Characterization of MCC@P-g-PCL biocomposite prepared

The MCC@P-g-PCL biocomposites were characterized by size exclusion chromatography (SEC), Fourier-transform infrared spectroscopy (FTIR), and phosphorus-31 nuclear magnetic resonance (^31^P NMR). SEC analysis was performed on the soluble free-PCL fraction recovered after extraction of the biocomposites. The molecular-weight characteristics of this extracted free-PCL fraction were used as an indirect estimate of the molecular weight of the PCL chains grafted onto MCC@P, and the corresponding results are summarized in Table 1. The number-average molecular weight (*M*_*n*_) of the extracted free PCL fraction decreased as the MCC@P content increased. Specifically, *M*_*n*_ significantly drops from 21.880 g mol^−1^ for MCC@P-g-PCL 1% to 8.312 g mol^−1^ for MCC@P-g-PCL 5%. This decrease in molecular weight with higher MCC@P loading can be attributed to the increased number of active sites available for *in situ* polymerization, which leads to more frequent chain initiation events and consequently shorter polymer chain lengths on average. The degree of polymerization (DP) was calculated from SEC results, correlates with the molecular weight data and confirms the expected polymer chain length. Indeed, a higher concentration of MCC@P enhances the density of active polymerization sites, thereby promoting the simultaneous formation of a higher number of polymer chains. Consistent with this rationale, the chain length tends to decrease as the MCC@P content rises, since the increased number of initiation sites accelerates chain formation and limits chain propagation. This observed trend aligns with previous reports in the literature, where *in situ* ring-opening polymerization (ROP) of ε-caprolactone conducted from various organic and inorganic fillers similarly demonstrated an inverse relationship between filler content and polymer chain length.^41,50,51^ Furthermore, the dispersity of the PCL chains of the prepared biocomposites was consistently below 1.6, suggesting that the polymerization may have occurred through a controlled process with minimal trans-esterification reactions.^52^ It worth mentioning that the relatively low molecular weight of the obtained polymer significantly affects both its mechanical and surface properties. From a mechanical standpoint, the reduced chain length leads to insufficient chain entanglement, resulting in poor cohesion, and increased brittleness. As matter of fact, the material lacks the structural integrity required to form stable and self-supported films. From a surface perspective, the high chain mobility associated with low molecular weight promotes surface rearrangement and increases sensitivity to solvents, which can compromise surface stability and reproducibility. These combined effects hinder the formation of uniforms, continuous films and limit the reliability of surface-related measurements such as swelling tests or contact angle analysis.

**Table 1:** The results obtained from SEC analysis for neat PCL and MCC@P-g-PCL biocomposites

	SEC measurements				
	*M*_*n*_ (g mol^−1^)	*M*_w_ (g mol^−1^)	*Đ*	DP	*G*_Y_%
Commercial-neat PCL	16 931	31 001	1.831	148	—
MCC@P-g-PCL 1%	21 880	35 877	1.64	192	46
MCC@P-g-PCL 3%	14 472	19 060	1.31	127	67.6
MCC@P-g-PCL 5%	8312	11 455	1.38	73	43.8

Moreover, the PCL grafting yield (*G*_Y_) was calculated. Indeed, *G*_Y_ for MCC@P-g-PCL (1%, 3% and) was increased from 46 to 67.6, which is due to the increase of phosphorus element; nevertheless, beyond 3% of MCC@P, an opposite effect could be stated due to the restriction effect of the polymerization growing chains.

On the other hand, the chemical structure of the MCC@P-g-PCL biocomposite prepared was investigated using Fourier transform infrared spectroscopy (FTIR). The spectra of MCC@P, neat PCL, and MCC@P-g-PCL biocomposites are overlaid in Fig. 3. In the FTIR spectrum of phosphorylated microcrystalline cellulose (MCC@P), a broad absorption band observed between 3200-3400 cm^−1^ is attributed to hydroxyl groups.^40^ An additional peak around 1280 cm^−1^ is generally attributed to the stretching vibration of P=O bond.^53^ Complementarily, ICP-OES analysis revealed a degree of substitution (DS) of 0.029, indicating an efficient incorporation of phosphate content introduced into the MCC backbone. Besides, FTIR spectrum of neat PCL displays characteristic peaks at 2945 cm^−1^ and 2865 cm^−1^, corresponding to the stretching vibrations of CH_2_ and at 1725 cm^−1^, attributed to the stretching vibration of the carbonyl groups. As shown in Fig. 3, the FTIR spectra of MCC@P-g-PCL biocomposites show a fingerprint of neat PCL. In addition, the noticeable decrease in the intensity of the hydroxyl groups absorption band intensity after graft copolymerization is also evident, indicating the transformation of several hydroxyl groups into polymerization (ROP) site of ε-CL.

**Fig. 3:**
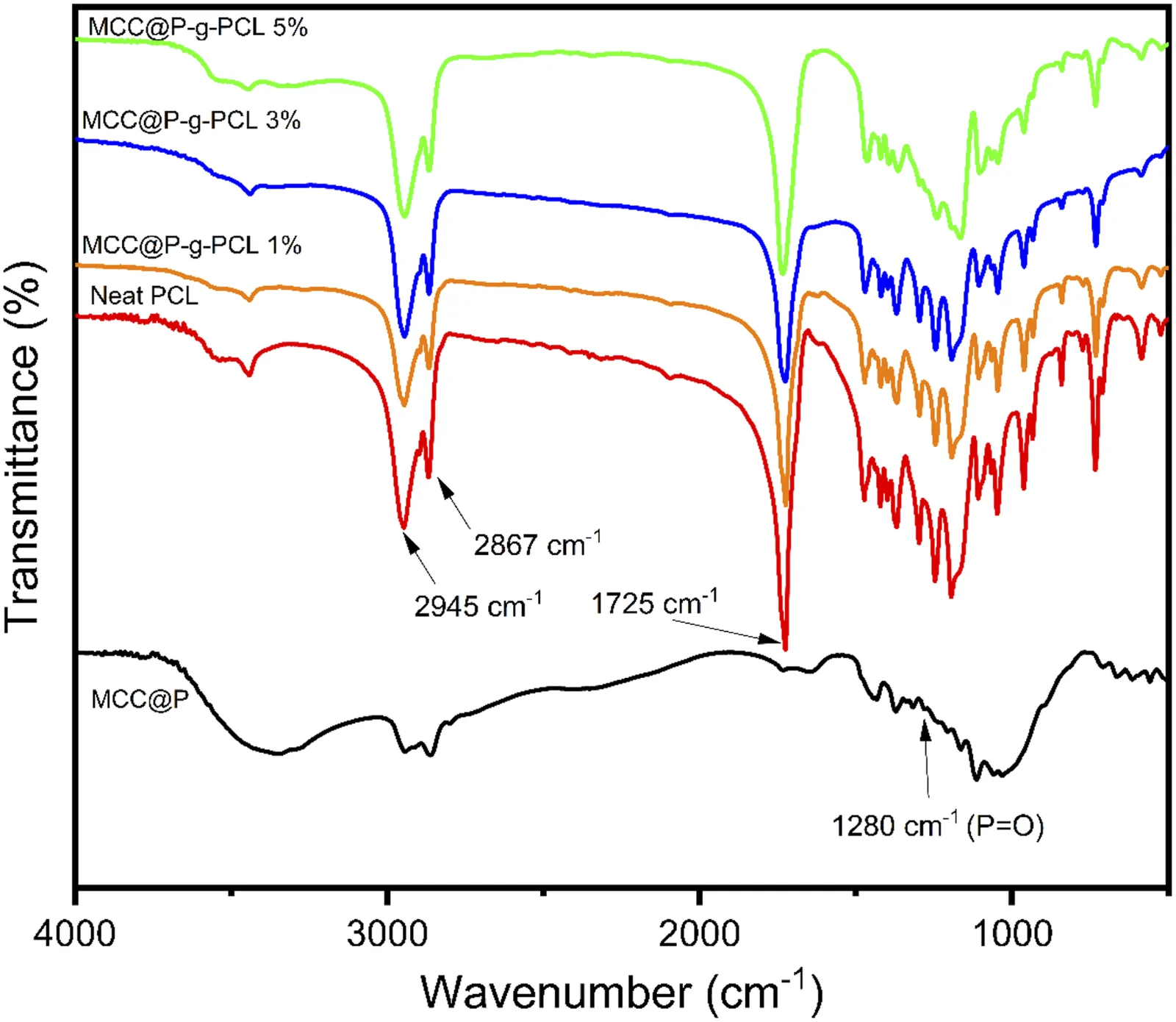
FTIR spectra of MCC@P, neat PCL, and MCC@P-g-PCL prepared biocomposites.

Solid-state ^13^C NMR initially investigated on MCC and MCC@P, confirming that MCC@P maintained the cellulose backbone with subtle shift (Fig. 4). To confirm the successful phosphorylation of MCC, ^31^P NMR spectroscopy has been performed on the prepared MCC@P (Fig. 5). A distinct signal was observed at −0.47 ppm, corresponding to the grafted phosphorus moiety. This chemical shift is notably different from that of unreacted POCl_3,_ which typically resonates at 2.6 ppm, confirming the effective incorporation of the phosphorus group onto the MCC backbone.

**Fig. 4:**
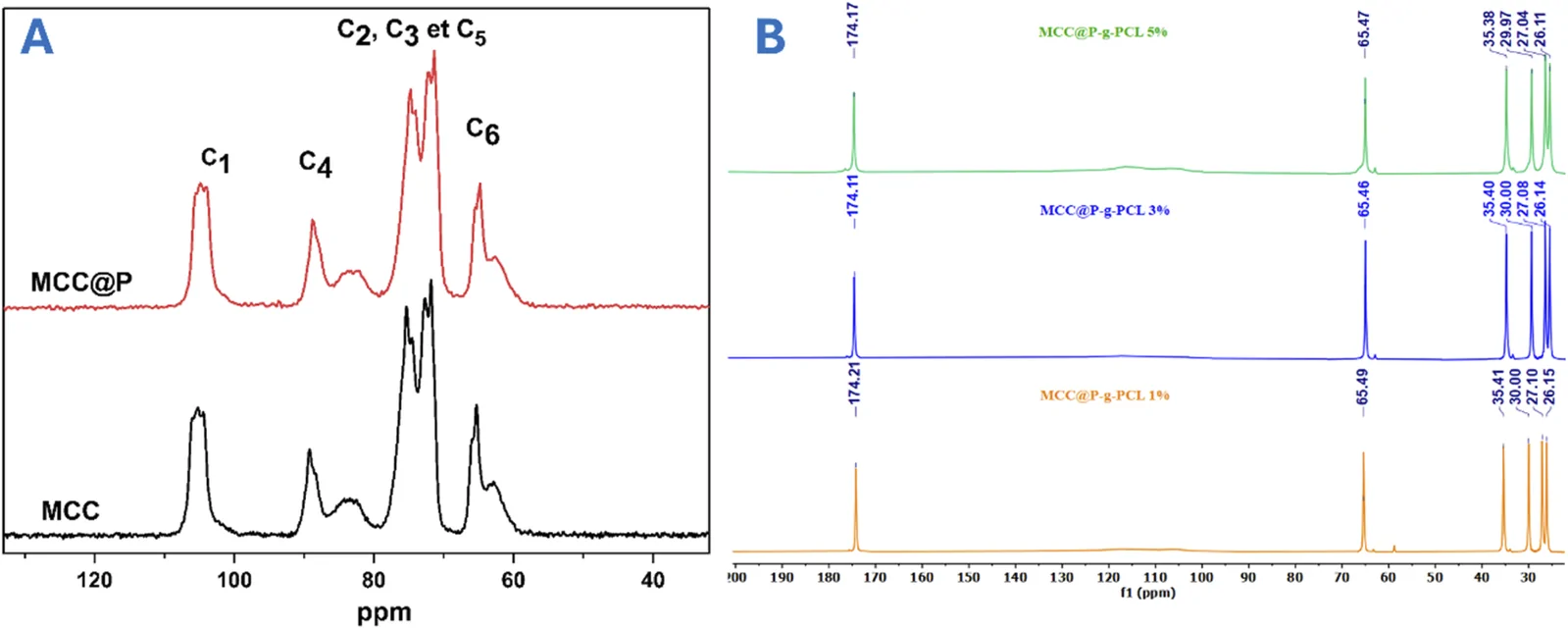
^13^C CP MAS NMR of (A) MCC and MCC@P; (B) MCC@P-g-PCL biocomposites.

**Fig. 5:**
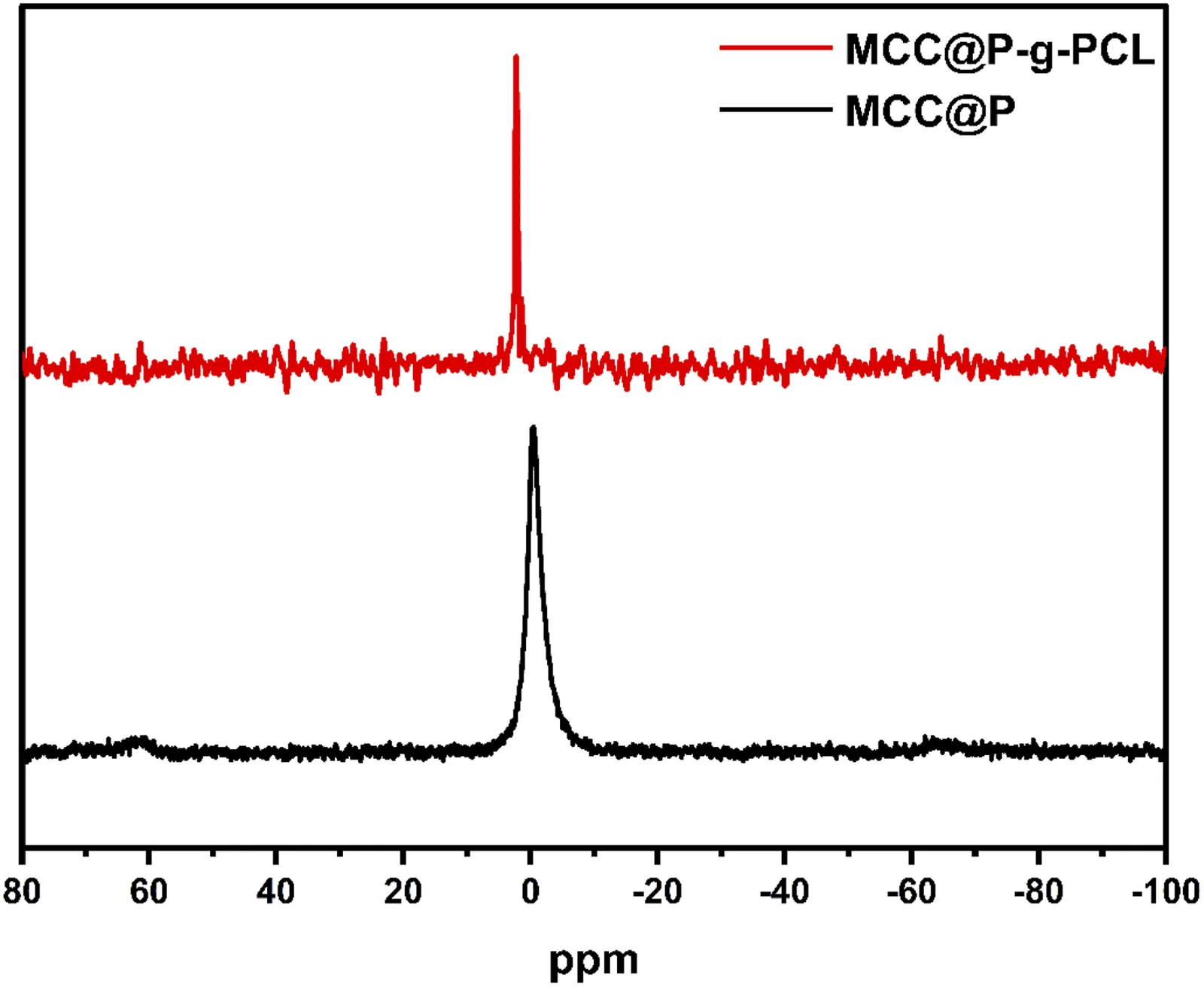
^31^P NMR spectra of MCC@P and MCC@P-g-PCL composite.

Compared to the signal observed at 0.47 ppm in MCC@P, a downfield shift to 2.16 ppm was detected in the spectrum of MCC@P-g-PCL (Fig. 5). This noticeable change in chemical shift reflects a modification in the electronic environment surrounding the phosphoryl group, likely resulting from its involvement in the grafting reaction. These results provide clear evidence for the successful covalent attachment of PCL chains onto the phosphorylated MCC backbone *via in situ* ring-opening polymerization (ROP) of ε-caprolactone. Finally, solid-state ^13^C NMR of MCC@P-g-PCL biocomposites, the ^13^C NMR (Fig. 4) spectra were largely governed by PCL peaks, while the low cellulose content rendered its signals nearly invisible. However, the ^31^P NMR spectra verified that MCC@P remained present in the final biocomposite.

Dynamic light scattering (DLS) technique was performed to determine the average particle sizes of phosphorylated microcrystalline cellulose (MCC@P) and the prepared biocomposites (MCC@P-g-PCL) that had been dispersed in THF. As shown in Table 2, the average particle size increased clearly, rising from 2913 nm for MCC@P to 4504 nm for MCC@P-g-PCL 3%. This increase in apparent particle size may be associated with surface modification by PCL and/or changes in particle aggregation and dispersion behavior. The observation is therefore considered as complementary evidence supporting the proposed grafting, rather than as direct proof of covalent attachment. In line with previous work,^54,55^ the grafting of PCL onto polysaccharides has been consistently correlated with an increase in particle size, as determined by DLS analyses.

**Table 2:** Averaged particles size obtained from DLS

Samples	Averaged size (nm)
MCC@P	2913
MCC@P-g-PCL 1%	3070
MCC@P-g-PCL 3%	4504
MCC@P-g-PCL 5%	2083

In addition to FTIR and NMR characterizations, suspension stability was carried out to assess the grafting success of the PCL chains from the phosphorylated microcrystalline cellulose. In this part, dichloromethane (CH_2_Cl_2_) was used as a non-solvent of MCC@P. The prepared biocomposite MCC@P-g-PCL, the physical mixture of MCC@P/PCL, and MCC@P were mixed in CH_2_Cl_2_. After two hours, the resulting suspensions stability of the samples was compared, as shown in Fig. S1 SI. For MCC@P and its physical mixture with PCL, sediment deposition was rapidly observed related to the non-solubility of MCC@P in CH_2_Cl_2_. In turn, the synthesized biocomposite MCC@P-g-PCL suspension remained stable in such non-polar solvent (CH_2_Cl_2_) indicating that PCL chains were able to keep the hydrophilic side (MCC@P) in a stable suspension. This behavior provides additional, indirect support for the proposed PCL grafting and improved interfacial compatibility.

TGA analysis was performed to assess and compare the thermal stability of neat PCL and the prepared biocomposites (MCC@P-g-PCL). Fig. 6 compares the thermal stability by superposing TGA curves of the prepared MCC@P-g-PCL (1, 3, and 5%) biocomposites and the neat PCL. It can be clearly observed that the thermal stability of MCC@P-g-PCL composite materials was enhanced compared to the neat PCL polymer, indicating that the incorporation of MCC@P as a filler significantly affected the thermal stability of the biocomposites prepared. The temperatures corresponding to 30% weight loss (*T*_d_) were 394 °C, 384 °C, and 370 °C for MCC@P-g-PCL 1%, MCC@P-g-PCL 3%, and MCC@P-g-PCL 5%, respectively. This gradual decrease in thermal stability is consistent with the decrease in the estimated molecular weight of the prepared biocomposites, from 21 880 to 14 472 and 8312 g mol^−1^, respectively, which may contribute to their lower thermal resistance. A similar trend has been reported in previous PCL-based biocomposites.^41,51,56^ Moreover, in contrast to neat PCL, which demonstrated complete degradation at around 510 °C, the prepared biocomposites MCC@P-g-PCL 1%, 3%, and 5% exhibited residual values of 0.8, 1.6, and 1.4, respectively. This variation can potentially be explained by the considerable thermal resistance of MCC@P as organic filler at this range of temperature <600 °C.^53,57^

**Fig. 6:**
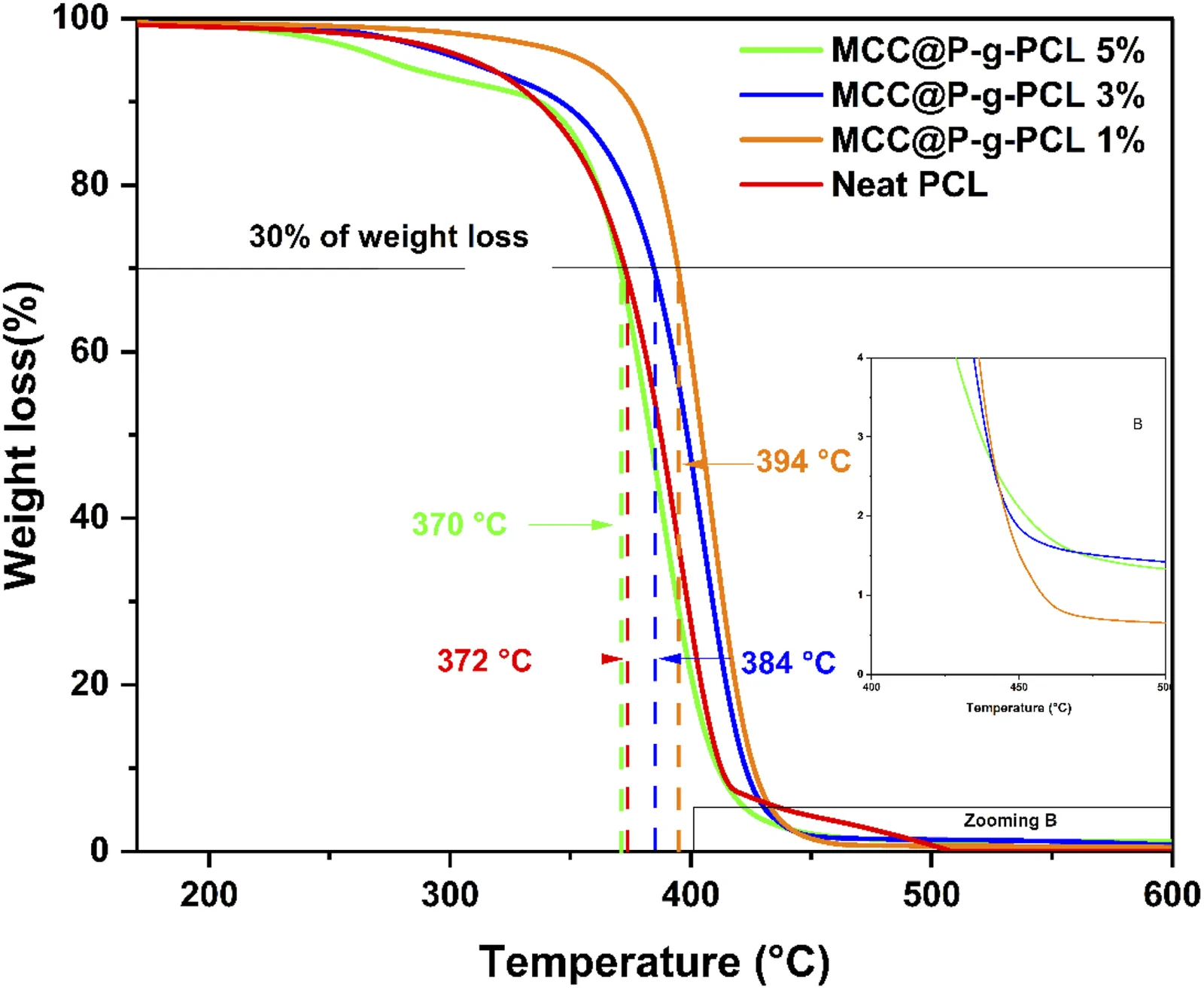
TGA curves of neat PCL and MCC@P-g-PCL (1%, 3%, and 5%) composites.

The second thermal investigation has been carried out by exploiting results obtained from the differential scanning calorimetry (DSC) analyses. In fact, the effects of MCC@P on PCL biocomposites' melting and crystallization behavior were investigated. The first cooling and second heating scans are presented in Fig. 7a and b, respectively. From Fig. 7a, the cooling curve of the MCC@P-g-PCL composite corresponds to PCL crystallization. Fig. 7 depicts the subsequent heating scan and facilitates the identification of PCL's melting point. Table 3 summarizes the results collected from the DSC curves of neat PCL and the prepared biocomposites (MCC@P-g-PCL). The melting point (*T*_m_), and crystallization temperature (*T*_c_) of the synthesized composites were compared to those obtained from neat PCL. In fact, the crystallization temperatures of the prepared composites, it has been noticed that *T*_c_ was higher than the value of the neat PCL. Precisely, the *T*_c_ value increased from 25.54 °C for neat PCL to 34.08 °C for MCC@P-g-PCL 1%, this increase in *T*_c_ could also be attributed to the difference in molecular weight between neat PCL and MCC@P-g-PCL composites. On the other hand, the crystallinity of PCL chains in neat and PCL-based biocomposites prepared was also calculated and compared. The degree of crystallinity (*χ*_c_%) of the PCL phase was also calculated from the DSC data and compared with that of neat PCL. As shown in Table 3, the crystallinity of the composites was higher than that of neat PCL, with *χ*_c_ values of 64.83%, 62.98%, and 60.76% for MCC@P-g-PCL 1%, 3%, and 5%, respectively, compared with 52.11% for neat PCL. This increase in crystallinity may be related to the influence of the MCC@P phase on the organization and crystallization of the PCL chains. The variation in PCL molecular weight with increasing MCC@P content may also contribute to the observed differences in crystallization behavior. The melting temperatures (*T*_m_) remained relatively close to that of neat PCL, ranging from 56.16 to 58.06 °C for the biocomposites, indicating that the incorporation of MCC@P did not substantially alter the melting behavior of the PCL phase.^58^

**Fig. 7:**
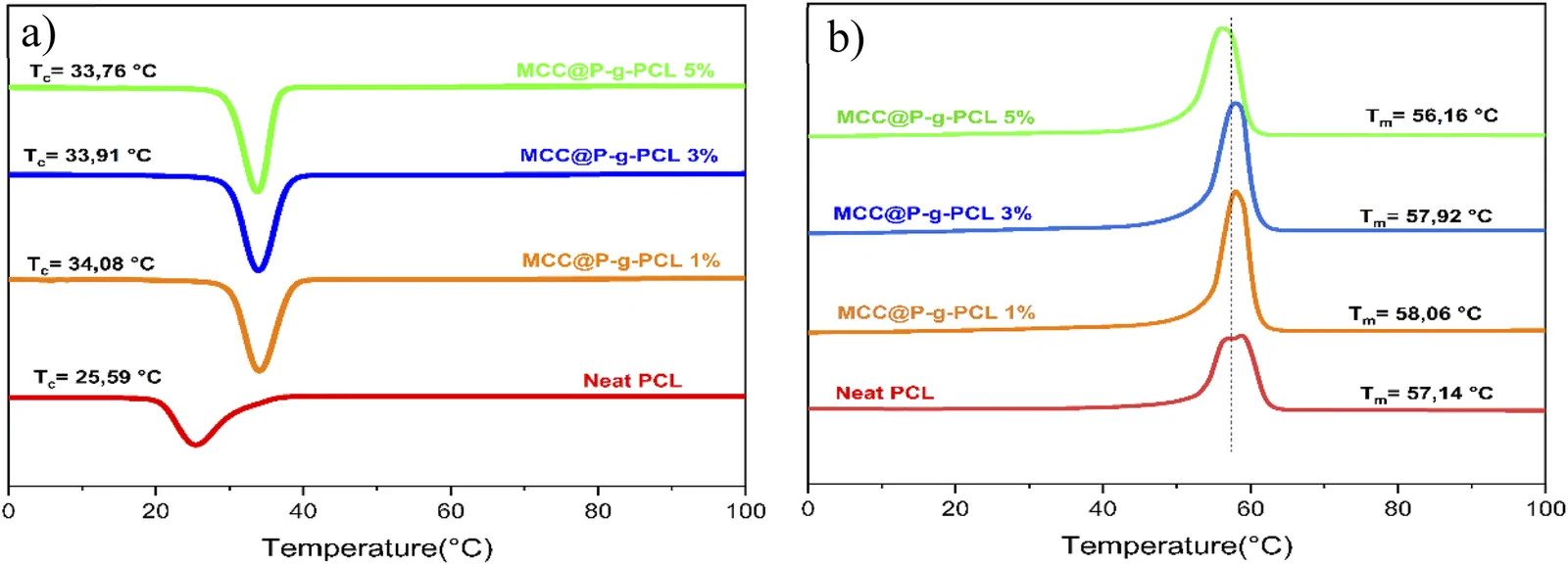
(a) Cooling and (b) heating DSC curves of MCC@P-g-PCL 1%, 3%, 5% and native PCL.

**Table 3:** DSC data of neat PCL and MCC@P-g-PCL nanocomposites with variable MCC@P content

	DSC measurements			
	*T*_m_ (°C)	*T*_c_ (°C)	Δ*H*_f_ (J g^−1^)	*χ*_c_ (%)
Neat PCL	57.14	25.54	71.08	52.11
MCC@P-g-PCL 1%	58.06	34.08	88.43	64.83
MCC@P-g-PCL 3%	57.92	33.95	85.91	62.98
MCC@P-g-PCL 5%	56.16	33.76	82.88	60.76

The surfaces of MCC, MCC@P, neat PCL, and PCL biocomposites (MCC@P-g-PCL) were examined by SEM to investigate their morphological characteristics (Fig. 8 and S2 SI). As revealed from the first SEM image (Fig. 8), MCC exhibits a smooth and consistent fibrous structure characteristic of its linear, unbranched polysaccharide chains. After phosphorylation, the images (Fig. 8) reveal subtle but clear alterations in the structure of cellulose: notably, this probably indicates that the functionalization did not alter the cellulose surface morphology. In contrast, neat PCL (Fig. 8) displays a smooth, uniform, and homogeneous texture. Upon incorporation of 1% of MCC@P into PCL matrix, the surface revealed small derbies of the polymer that were most probably formed during the polymerization process of the sample in addition to some dispersed microparticles surrounded by smooth PCL film that might be MCC@P (Fig. 8). For MCC@P-g-PCL 3% and 5% (Fig. S2 SI), the fractured surface showed irregularity, which may be due to the high amount of MCC@P incorporated.

**Fig. 8:**
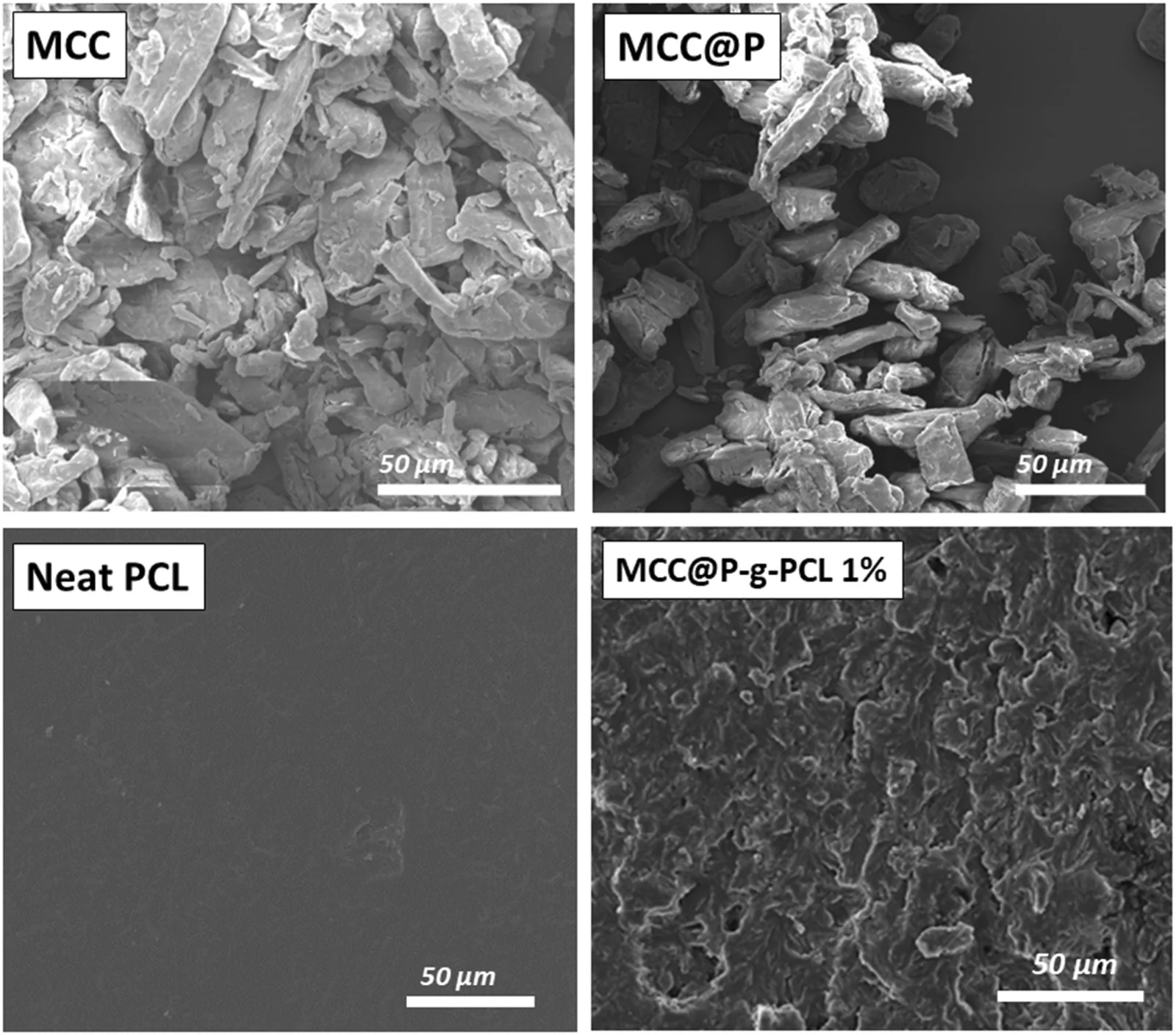
The SEM images of MCC, MCC@P, neat PCL and MCC@P-g-PCL 1%.

The XRD patterns of the neat MCC, MCC@P, neat PCL, and prepared PCL biocomposites are superposed in Fig. 9a and b and compared to investigate the effect of structural interactions and the degree of order of the prepared materials. Indeed, Fig. 9a shows the X-ray diffraction patterns of MCC and MCC@P; both exhibit the characteristic reflections of cellulose. They display three distinct crystalline peaks at 2*θ* values around 15°, 16.8°, 22.7°, and 34.6°, aligning with the (11̄0), (110), (200), and (004) crystallographic planes of cellulose.^50^ After phosphorylation, the intensity of the peaks remains broadly similar, indicating that the crystalline core of cellulose is broadly preserved. However, a slight decrease in intensity, particularly at 22.6°, of the intense (200) peak in the case of MCC@P suggests a partial disruption of the ordered crystalline domains, likely due to the introduction of phosphate groups on the cellulose chains. This modification seems to affect mainly the amorphous or surface regions, without fully disrupting the crystalline structure. Therefore, phosphorylation leads to a moderate reduction in crystallinity, while maintaining the typical architecture of cellulose. In Fig. 9b, neat PCL and the prepared composites (MCC@P-g-PCL) display two characteristic peaks at 23.75° and 21.45° corresponding to orthorhombic crystalline structures of PCL, which were indexed as (110) and (200) planes, respectively.^59^ Additionally, the prepared biocomposites (MCC@P-g-PCL) show similar peaks as neat PCL as well as an increase in the intensity of the peak at around 2*θ* = 22.7° due to the introduction of cellulose, which increased its intensity with an increasing amount of MCC@P. The increase in intensity 2*θ* around 22.7° with increasing MCC@P content is primarily attributed to the increasing contribution of the crystalline cellulose phase to the composite diffraction pattern. MCC@P may also act as a heterogeneous nucleating agent for PCL crystallization. However, because the diffraction contributions of cellulose and PCL overlap in this region, the increase in peak intensity alone cannot unambiguously demonstrate an increase in the intrinsic crystallinity of the PCL phase.^60,61^

**Fig. 9:**
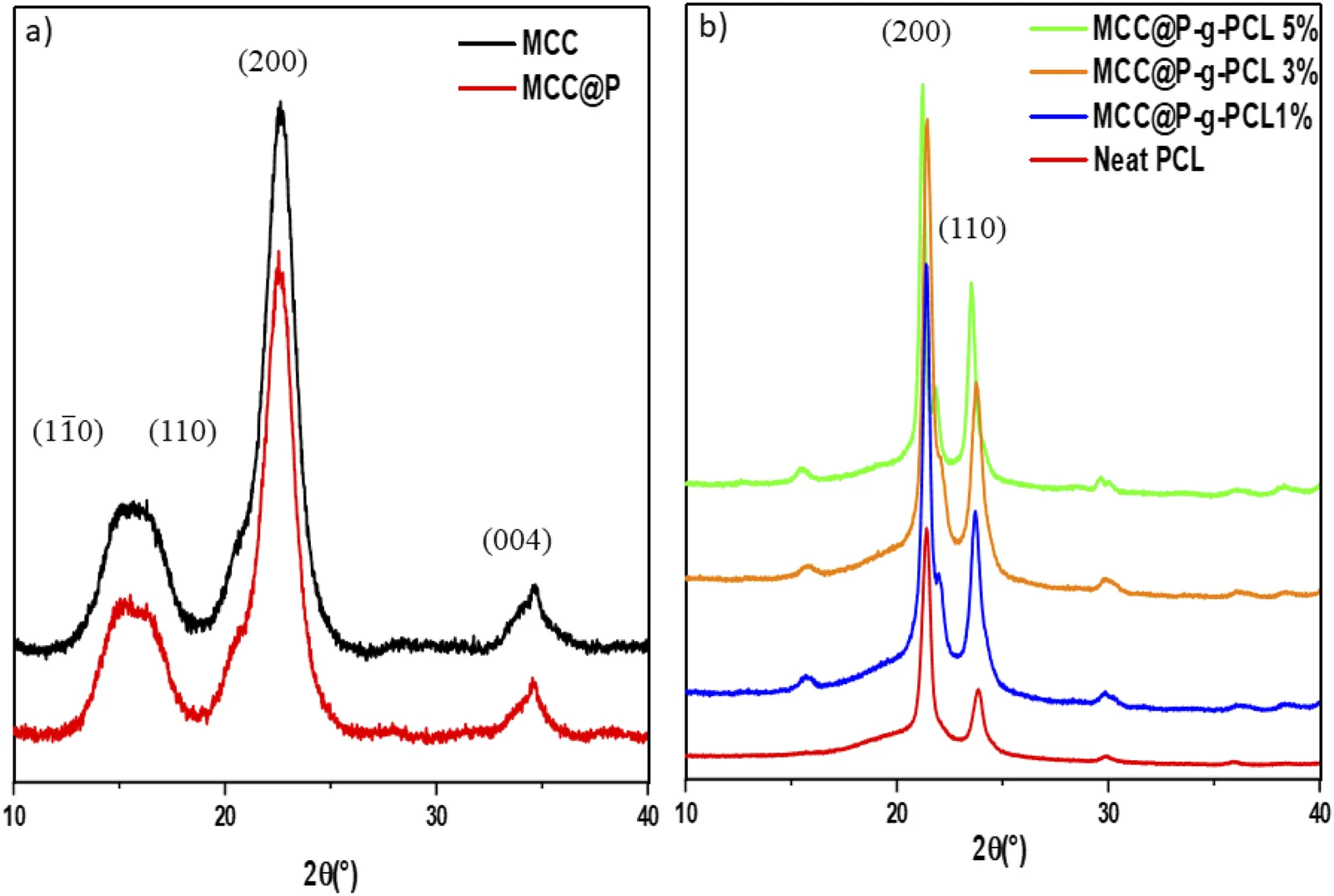
The XRD spectra of (a) MCC and MCC@P and (b) MCC@P-g-PCL 1%, 3%, 5% and native PCL.

The fire performance and thermal decomposition of the prepared MCC@P-g-PCL biocomposites were evaluated using a microscale combustion calorimeter, to assess the effectiveness of the phosphorus containing flame retardant materials. Essential assessment parameters such as heat release rate (HRR), total heat release (THR), and heat release capacity (HRC) were investigated to provide a numerical expression of the fire intensity.^62,63^ The experimental findings are graphically represented in Fig. 10 and summarized in Table S2 SI. The neat PCL sample shows high peak heat release (PHRR) and total heat release (THR) values of 694.25 W g^−1^ and 36.06 kJ g^−1^, respectively. In contrast, the PHRR and THR values were reduced with the increasing MCC@P content for the MCC@P-containing samples. Accordingly, using a small amount of phosphorylated MCC can decrease the PHRR and THR values, as observed from the experimental results. Compared with the neat PCL sample, the PHRR and THR values of the MCC@P-g-PCL 1% sample decreased to 648.71 W g^−1^ and 34.42 kJ g^−1^, which represent a reduction of 6.84% and 4.55%, respectively. Likewise, the heat release capacity (HRC) value of neat PCL (679.34 J g^−1^ K^−1^) was higher than that obtained with MCC@P-g-PCL biocomposites samples, which exhibited the lowest HRC value (656.93 J g^−1^ K^−1^, 556.41 J g^−1^ K^−1^ and 622.62 J g^−1^ K^−1^) for MCC@P-g-PCL 1%, 3% and 5% respectively, with an estimated reduction of 3.3%, 18.1% and 8.35% respectively. These results indicate that the incorporation of MCC@P modifies the heat-release behavior of the MCC@P-g-PCL biocomposites. In fact, the P element introduced into the cellulose chains acts in the condensed phase, forming a protective char layer against heat transfer.^64^ Overall, the incorporation of MCC@P reduced several heat-release parameters, with the largest reduction observed for the 3 wt% formulation. These results indicate an improvement in fire-related performance as measured by microscale combustion calorimetry.

**Fig. 10:**
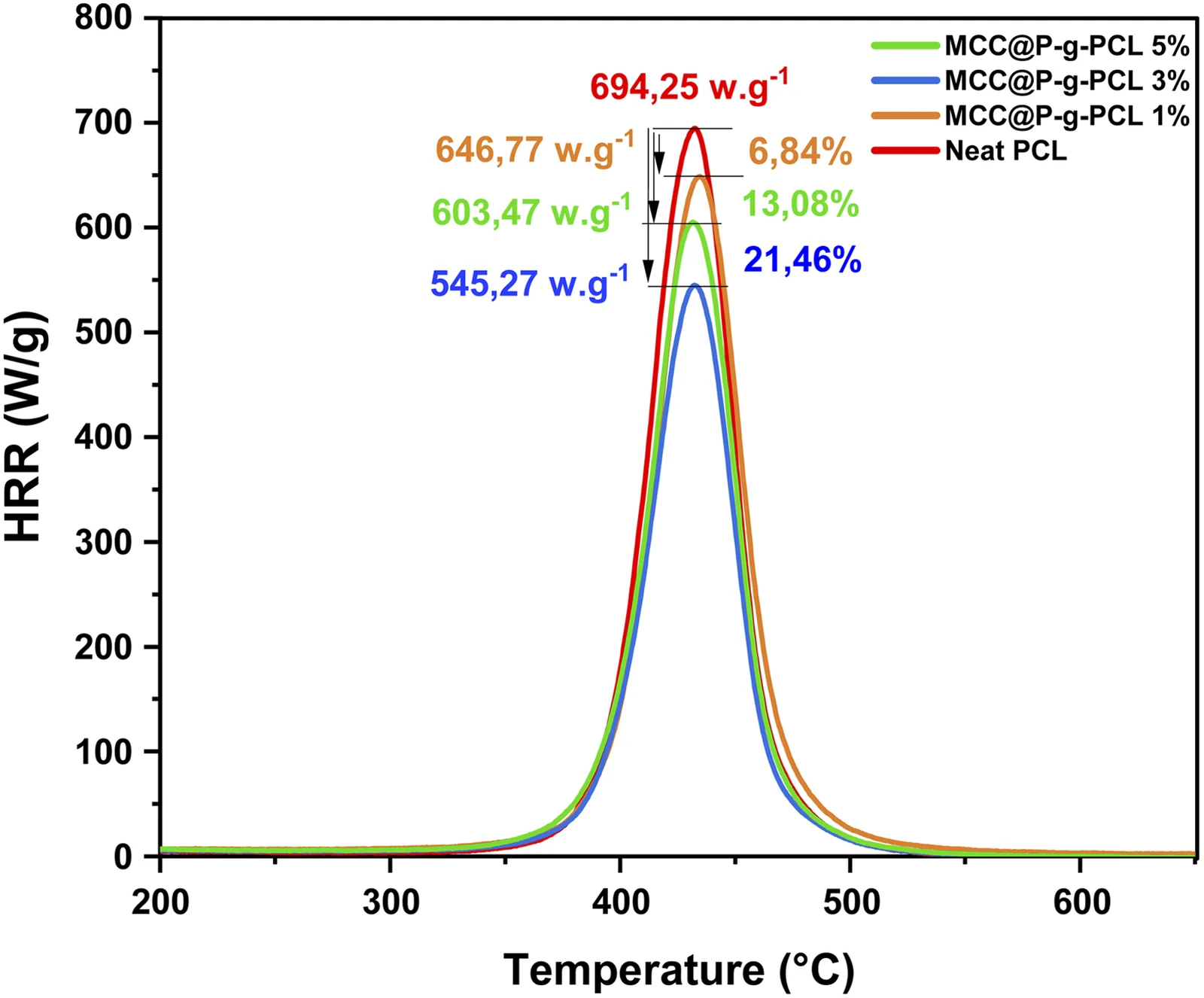
HRR as a function of temperature curves of neat PCL and MCC@P-g-PCL samples from the MsCC test.

Compared to our previous work on the Ti_3_C_2_T_*x*_@ADP-PCL MXene-based system.^65^ Where a more significant reduction in HRR was achieved due to the synergistic effect between MXene nanosheets (inorganic charge) and phosphorus functionalities, the MCC@P-g-PCL biocomposites exhibit a moderate yet meaningful improvement in fire performance. In the MXene-based system, the layered structure of Ti_3_C_2_T_*x*_@ADP provides an efficient physical barrier and smoke suppression effect leading to strong combustion inhibition. In contrast, the present system relies mainly on phosphorylated cellulose (MCC@P) as an intrinsic flame-retardant component. Although the reduction in heat release is less pronounced, the MCC@P-g-PCL approach offers the advantage of a fully biobased and more sustainable flame-retardant strategy.

## 4 Conclusion

This study demonstrates the promising potential of phosphorylated microcrystalline cellulose (MCC@P) as both a functional filler and a metal-free initiator for the ring-opening polymerization (ROP) of ε-caprolactone (ε-CL), enabling the synthesis of MCC@P-g-PCL biocomposites. The use of MCC@P enabled *in situ* bulk polymerization under metal-free and solvent-free conditions, and the combined characterization results support the proposed formation of MCC@P-g-PCL biocomposites. The findings underscore the multifunctional role of MCC@P, which not only facilitates polymer formation but also improves the thermal properties of the resulting MCC@P-g-PCL biocomposites. Comprehensive characterization through FTIR, ^31^P, ^13^C NMR spectroscopies, and size exclusion chromatography (SEC) supported the phosphorylation of MCC and provided complementary evidence consistent with the proposed grafting of PCL onto MCC@P. Compared with neat PCL, the prepared biocomposites (MCC@P-g-PCL) exhibited enhanced thermal stability and a narrower molecular weight distribution relative to neat PCL. Furthermore, the prepared PCL biocomposites also showed enhanced flame-retardant properties, as evidenced by the reduction in heat-release parameters compared with neat PCL, indicating improved thermal safety. Overall, these results demonstrate that phosphorylated MCC can serve as a multifunctional, metal-free initiator for the synthesis of PCL-based biocomposites with reduced dependence on organometallic catalysts and organic solvents.

## Author contributions

Hassan Ait Yachou: writing – original draft, methodology, formal analysis, data curation. El Hassan Boutriouia: writing, methodology, formal analysis, data curation. Taha El Assimi: writing, software, methodology, formal analysis, data curation. Sara Blilid: methodology, formal analysis, data curation. Abderrahim Maazouz: writing – review & editing, formal analysis. Hicham Ben Youcef: writing – review & editing, visualization, methodology, formal analysis. Khalid Lamnawar: writing – review & editing, validation, supervision. Abdelkrim El Kadib: writing – review & editing, supervision. Mohammed Lahcini: writing – review & editing, validation, supervision, resources.

## Conflicts of interest

There are no conflicts to declare.

## Supplementary Material

RA-016-D6RA07997K-s001

## Data Availability

The data underlying this study are available in the published article and the supplementary information (SI). Supplementary information is available. See DOI: https://doi.org/10.1039/d6ra07997k.
