# Supplementary material for: Dual synergistic role of phosphorylated microcrystalline cellulose as organoinitiator and filler for PCL biocomposites
Source: RSC Adv. 2026 Sep 30;16(46):59374–87. doi: 10.1039/d6ra07997k (PMC13625057; doi:10.1039/d6ra07997k)
Supplement: Supplementary file 1 [file RA-016-D6RA07997K-s001.pdf]

Supplementary Information (SI) for RSC Advances.
This journal is © The Royal Society of Chemistry

Dual Synergistic Role of Phosphorylated Microcrystalline Cellulose as Organo-catalyst and Filler for PCL Biocomposites

Hassan Ait Yachou [1,2], El Hassan Boutriouia [1,3], Taha El Assimi[1,3], Sara Blilid [4], Abderrahim Maazouz [2,5], Hicham Ben Youcef [3], Khalid Lamnawar [2], Abdelkrim El Kadib [4], Mohammed Lahcini[1,3].

[1] IMED-Lab, Faculty of sciences and techniques, Cadi Ayyad University (UCA), Avenue Abdelkrim Elkhattabi, B.P 549, 40000 Marrakech, Morocco.

[2] Univ Lyon, CNRS, UMR 5223, Ingénierie des Matériaux Polymères, INSA Lyon, Université Claude Bernard Lyon 1, Université Jean-Monnet, F-69621 Villeurbanne, France.

[3] College of Chemical Sciences and Engineering (CCSE), Mohammed VI Polytechnic University (UM6P), Benguerir 43150, Morocco.

[4] Euro-Med University of Fes (UEMF), Route de Meknès, Rond-point de Bensouda, 30070, Fez, Morocco.

[5] Hassan II Academy of Science and Technology, Rabat - Morocco.

Supporting information

Experimental section.

Table 1SI. Phosphorus content and calculated degree of substitution (DS) of phosphorylated MCC.

Figure 1SI. Suspension stability test; A, B, C and A',B',C' are the physical mixture (MCC@P/PCL), MCC@P-g-PCL biocomposite and MCC@P in dichloromethane before and after 2 hours , respectively.

Figure 2SI. SEM images of neat and prepared PCL biocomposites

Table 2SI. Data of Neat PCL and MCC@P-g-PCL samples from the microscale combustion calorimeter analysis

Table 1SI. Phosphorus content and calculated degree of substitution (DS) of phosphorylated MCC.

Sample	P content (wt%)	Method	DS
MCC@P	0.55	ICP-OES	0.029

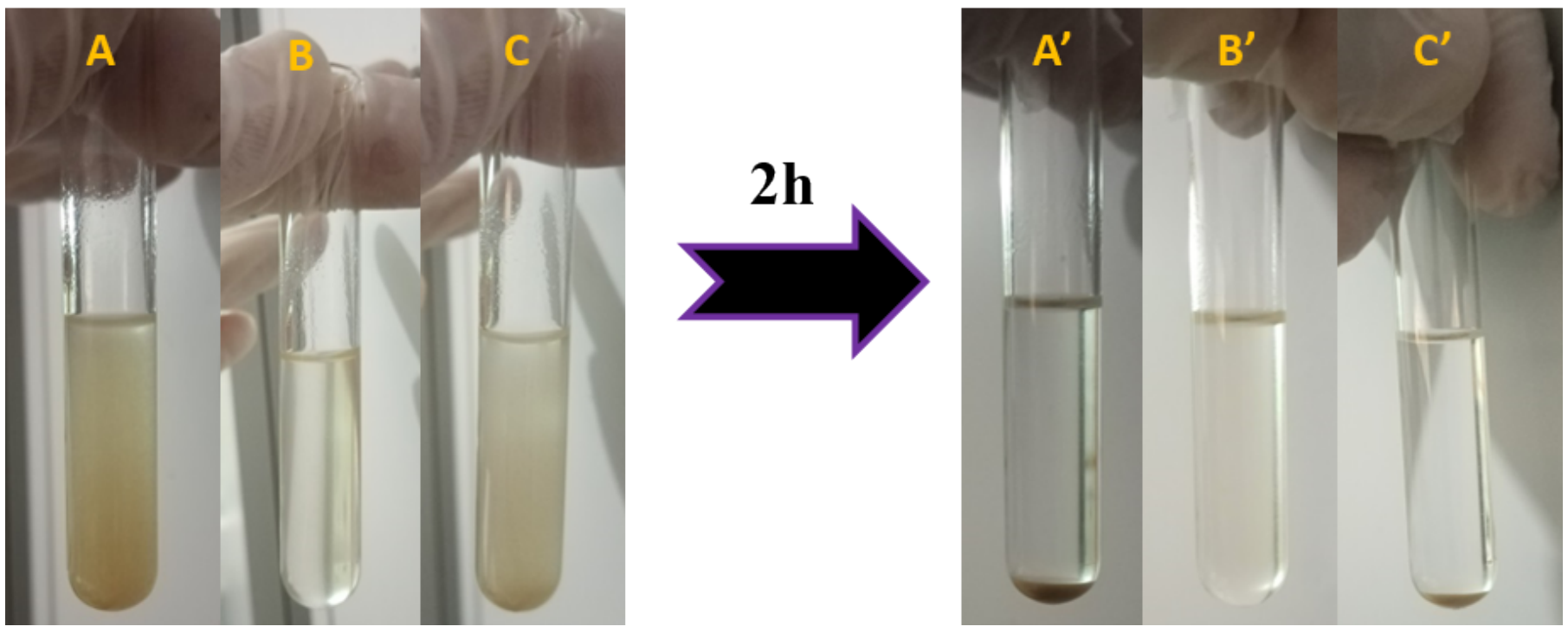

Figure 1SI. Suspension stability test; A,B,C and A',B',C' are the physical mixture (MCC@P/PCL),MCC@P-g-PCL biocomposite and MCC@P in dichloromethane before and after 2 hours , respectively.

Table 2S1. Data of Neat PCL and MCC@P-g-PCL samples from the microscale combustion calorimeter analysis

Samples	*HRR ($W\ g^{-1}$)*	*THR* ($KJ\ g^{-1}$)	*HRC ($J\ g^{-1}\ K^{-1}$)*
Neat PCL	694.86	36.06	679.34
MCC@P-g-PCL 1%	648.71	34.42	656.93
MCC@P-g-PCL 3%	545.28	28.84	556.41
MCC@P-g-PCL 5%	605.19	33.05	622.62

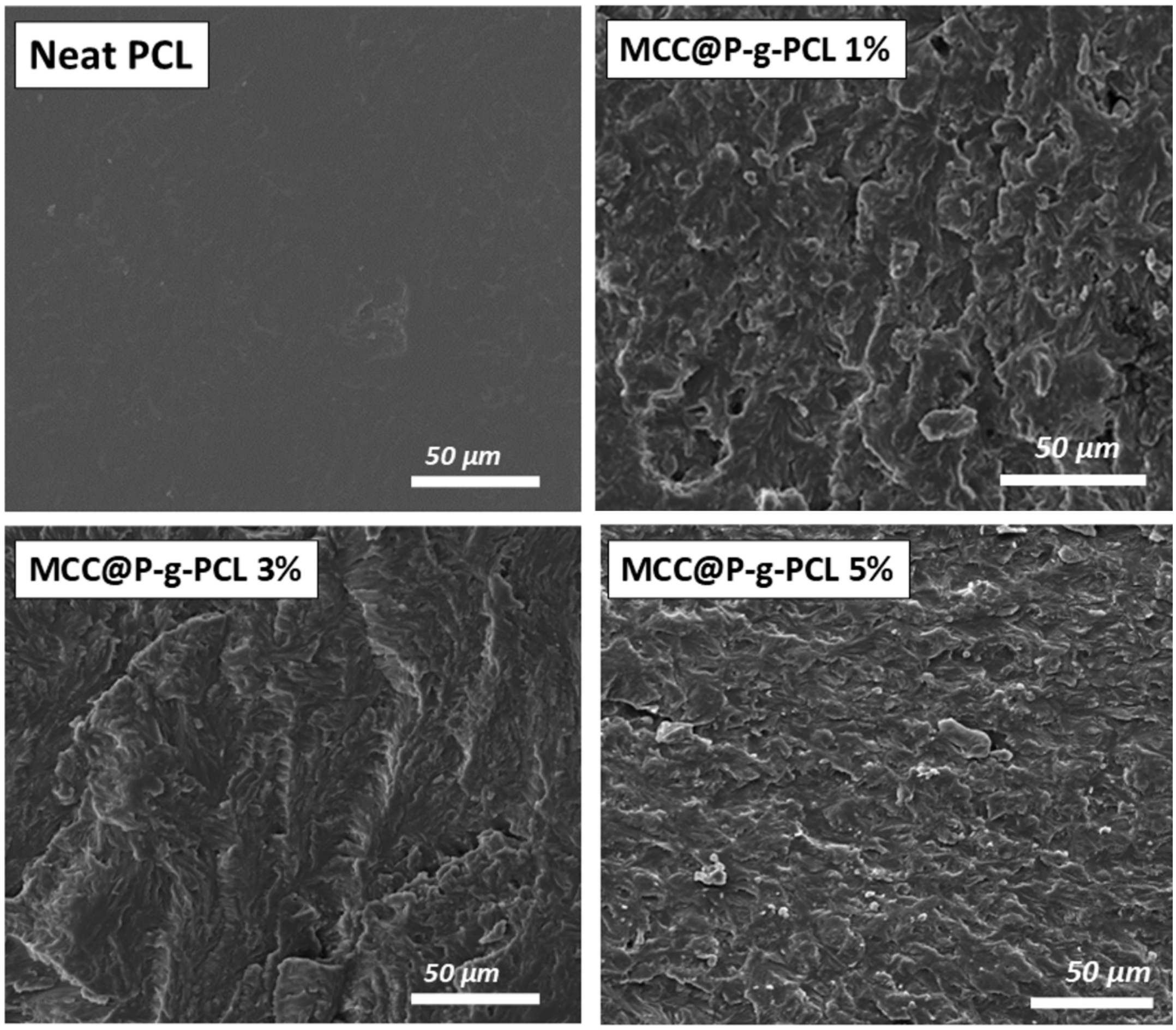

Figure 2SI. SEM images of neat and prepared PCL biocomposites.
